# Energy drink use and its health implications among medical students in Aden, Yemen: a cross-sectional study

**DOI:** 10.1186/s12889-026-28380-3

**Published:** 2026-07-10

**Authors:** Hanan A. A. Mohammed, Ali N. M. Gubran, Sara Mohammed Ali Kolais, Ayah Mohammed Omar, Safia Ghaleb Zain Salem

**Affiliations:** Department of Health Sciences, Faculty of Medicine and Health Sciences, University of Science and Technology, Aden, Yemen

**Keywords:** Energy drinks, Health implications, Medical students

## Abstract

**Background:**

The consumption of energy drinks (EDs) is becoming an increasingly common habit among university students, especially those in demanding medical fields. This study aimed to determine the prevalence of energy drink consumption among medical university students in Aden, Yemen.

**Methods:**

A cross-sectional study was conducted among 400 university students from both public and private universities between May and August 2025. Data were collected using a structured questionnaire and analyzed using SPSS.

**Results:**

The overall prevalence of ED consumption was 62.5%. Consumption was higher among males (68.5%) and younger students aged ≤ 19 years (73.6%), and both showed a statistically significant association (*p* < 0.05). ED consumption was higher in the evening (59.2%), and the most commonly consumed brand was Lift Up. Academic pressure was a major contributing factor, as 76.8% of respondents reported increased intake during examinations. Nearly half of the participants (44.8%) reported combining energy drinks with other substances. Although 83.8% believed that EDs improve concentration and energy, most also reported adverse effects, particularly insomnia (66%), anxiety or stress (58.8%), heart palpitations (58.2%), and dehydration (58.4%). Significant associations were found between ED consumption and daily coffee consumption as well as qat chewing (75.7% and 64.9%, *p* = 0.017 and *p* = 0.007), respectively.

**Conclusion:**

Energy drink consumption is highly prevalent among medical students in Aden and is influenced by demographic and academic factors. Despite widespread awareness of potential adverse health effects, consumption remains driven by perceived academic benefits. These findings highlight the need for university-based health promotion programs and targeted educational interventions to increase awareness of the risks associated with energy drink use and to encourage healthier coping strategies during academic stress.

## Introduction

Energy drinks (EDs) can be described as one of the most widely consumed drinks of the last decades all over the world [1, 2]. Since their appearance, they have turned into a very serious consumerist phenomenon among adolescents and young adults [3, 4]. The energy drinks are made with high levels of caffeine and also contain other substances like taurine, B vitamins, sugar, and amino acids [5, 6], whose supplementation is claimed to enhance alertness, to increase energy, and to improve physical and cognitive performance [7, 8]. The aggressive marketing strategies and accessibility of EDs have contributed to their growing consumption, especially among university students who are exposed to academic pressures and a highly competitive environment [9, 10, 11, 12]. Survey studies have shown that the rates of energy drink consumption are particularly high among adolescents and university students, who represent a key market segment for these products. [13, 14, 15, 16, 17, 18, 19]. In the competitive academic environment, university students have to cope with increasing pressures that demand long hours of study, preparation for exams, and the need to balance social and academic activities [1, 5]. As a result, a significant number of university students have turned to EDs as a convenient and quick solution to alleviate fatigue, enhance concentration during long night study sessions, and enhance academic performance [2, 9]. Studies have found that the highest usual causes for the consumption of energy drinks by university students include the need for energy due to lack of sleep [1, 2]. Additionally, social influences, peer pressure, and participation in parties or athletic activities often encourage energy drink use, sometimes in combination with other substances such as Qat or Shammah Al-Hot products (a locally used smokeless tobacco product) [20, 21 , 22]. Qat is an evergreen shrub belonging to the family *Celastraceae*, traditionally consumed in Yemen and countries of the Horn of Africa, where chewing its fresh leaves is a common cultural practice [23]. Despite these identified factors for consumption, research findings suggest that heavy EDs consumption is associated with negative health and well-being outcomes for students [24, 25, 26, 27, 28]. Research findings have also identified the consumption of EDs with negative effects on the sleep patterns of consumers, such as reduced quality and quantity of sleep, resulting in a cycle of caffeine dependence for consumers of EDs [9, 10, 11]. Additionally, there is an emerging body of research that identifies negative effects on the psychology of consumers of EDs, with research findings indicating that consumption of energy drinks is linked with increased anxiety and distress among student populations. In some cases, overuse of these products has also been associated with more serious mental health concerns, further emphasizing the importance of closely monitoring their effects on young adults [12, 13, 22]. Despite growing global concerns regarding the adverse effects of energy drinks, research within the Yemeni academic context remains limited, particularly in Aden as temporary capital of Yemen, which was selected as the study setting due to its role as a major academic center in Yemen and the lake previous studies on energy drink consumption among its medical students. This study addresses an important gap in the literature as one of the first of its kind in this city, specifically targeting medical and health sciences students. This population is of unique interest; while they represent the future of the healthcare system, the rigorous nature of their studies may predispose them to high consumption patterns. This research aimed to determine the prevalence of energy drink consumption among university medical students in Aden, Yemen, and to assess their knowledge of health risks and effects of EDs.

## Subjects and methods

### Study design

The study design was a cross-sectional descriptive survey to assess the consumption patterns of energy drinks among medical college students.

### Study area and duration

The study was carried out in different universities and colleges across Aden, including both public and private universities. The data collection process took place over a period of three months, from the 10th of May to the 6th of August 2025.

### Sample size and sampling method

Cochrane’s formula was used to calculate the sample size using a previously reported prevalence of 42.2% from a Libyan study, representing the overall prevalence of energy drink consumption (30% regular and 12.2% occasional consumers) [29], a 95% confidence level, and a margin of error of 5%.

$$n=\:\frac{{{z}}^{2}\times\:{p}{\%}(1-{p}{\%})\:}{{\mathrm{d}}^{2}}$$

Where**n** is the required sample size, z represents the z-score, p represents the estimated prevalence of the outcome of interest, and d represents the margin of error.

$$\begin{aligned}\:\frac{{(1,96)}^{2}\times\:0.422(1-0.422)}{{(0,05)}^{2}}\:\mathbf{n}\;&=\;\frac{3.84\times\:0.244}{0.0025}\\&=375\:\:{Students}\end{aligned}$$

According to Cochran’s formula, the minimum sample size that should be used is 375 students. However, considering that some students might not respond to the questionnaire, a higher number of students were given the questionnaire. A total of 400 students were included. A stratified sampling technique was used to recruit medical students from both public and private universities in Aden.

### Inclusion and exclusion criteria

In this study, male and female students who were enrolled in any year level in medical-related colleges (medicine, dentistry, pharmacy, nutrition, radiology, and medical laboratory science) and who are willing to participate in this research were considered. Only students aged 18 years and above were included in the study. In Yemen, students generally enter university after completing a relatively long educational pathway, typically at the age of 18 years or older; therefore, all participants in this study were adults and eligible to provide informed consent. The exclusion criteria were non-medical students, unwillingness to participate in this research, and incomplete answers in the questionnaires.

### Data collection tools and procedures

The data collection tool was a structured questionnaire adapted from previously published and validated studies on similar topics [1, 30]. The questionnaire consisted of 15 items and was divided into three main sections. The first section included socio-demographic characteristics such as age, gender, area of study, and year of study. The second section assessed participants’ energy drink consumption behaviors, including frequency of use, preferred brands, time of consumption, and reasons for use, as well as co-consumption with other substances. Consumption frequency was categorized as daily, often, and sometimes, where “often” referred to regular but not daily consumption, and “sometimes” referred to occasional or irregular use as self-reported by participants. The third section evaluated participants’ knowledge and perceptions regarding energy drinks, including perceived effectiveness, health concerns, and self-reported adverse effects such as anxiety, tachycardia, insomnia, dehydration, and weight gain.

The questionnaire required approximately 5–10 min to complete. It was administered in English, with clarification provided in Arabic when necessary to ensure understanding among participants. The questionnaire was adapted from previously validated instruments, and its content validity was reviewed by subject experts. A pilot study was conducted to ensure clarity and appropriateness of the items before data collection.

### Data analysis

The collected data was entered and analyzed using computer software (SPSS version 25) for analysis. Descriptive statistics were used to describe the variables of interest. The descriptive statistics used were frequency, percentage, mean, and standard deviation. relationship between variables was also analyzed using the chi-square test for association. The level of statistical significance was set at 0.05 for this study. Before conducting the analysis, the completeness and consistency of the collected data were evaluated. Data that were incomplete were excluded from further analysis.

## Results

A total of 400 university students participated in the study. The mean age of participants was 21.53 ± 2.77 years. Nearly half of the respondents (48.0%) were aged 20–24 years. Males accounted for 55.5% of the sample, and the vast majority were unmarried (97.0%). Regarding academic characteristics, third-year students represented the largest proportion (28.7%), while medicine was the most common field of study (24.0%) (Table 1).

**Table 1:** Demographic characteristics of participants (*n* = 400)

Category	Frequency (*n*)	Percentage (%)
Age		
Mean ± SD (21.53 ± 2.774)		
≤ 19	106	(26.5%)
20–24	192	(48.0%)
≥ 25	102	(25.5%)
Sex		
Males	222	(55.5%)
Female	178	(44.5%)
Marital Status		
Unmarried	388	(97.0%)
Married	12	(3.0%)
Field of Study		
Medical laboratories	78	(19.5%)
Nutrition	53	(13.3%)
Pharmacy	60	(15.0%)
Dentistry	91	(22.8%)
Medicine	96	(24.0%)
Radiology	22	(5.5%)
Year of Study		
First	75	(18.8%)
Second	107	(26.8%)
Third	115	(28.7%)
Fourth or more	103	(25.8%)

**Fig. 1:**
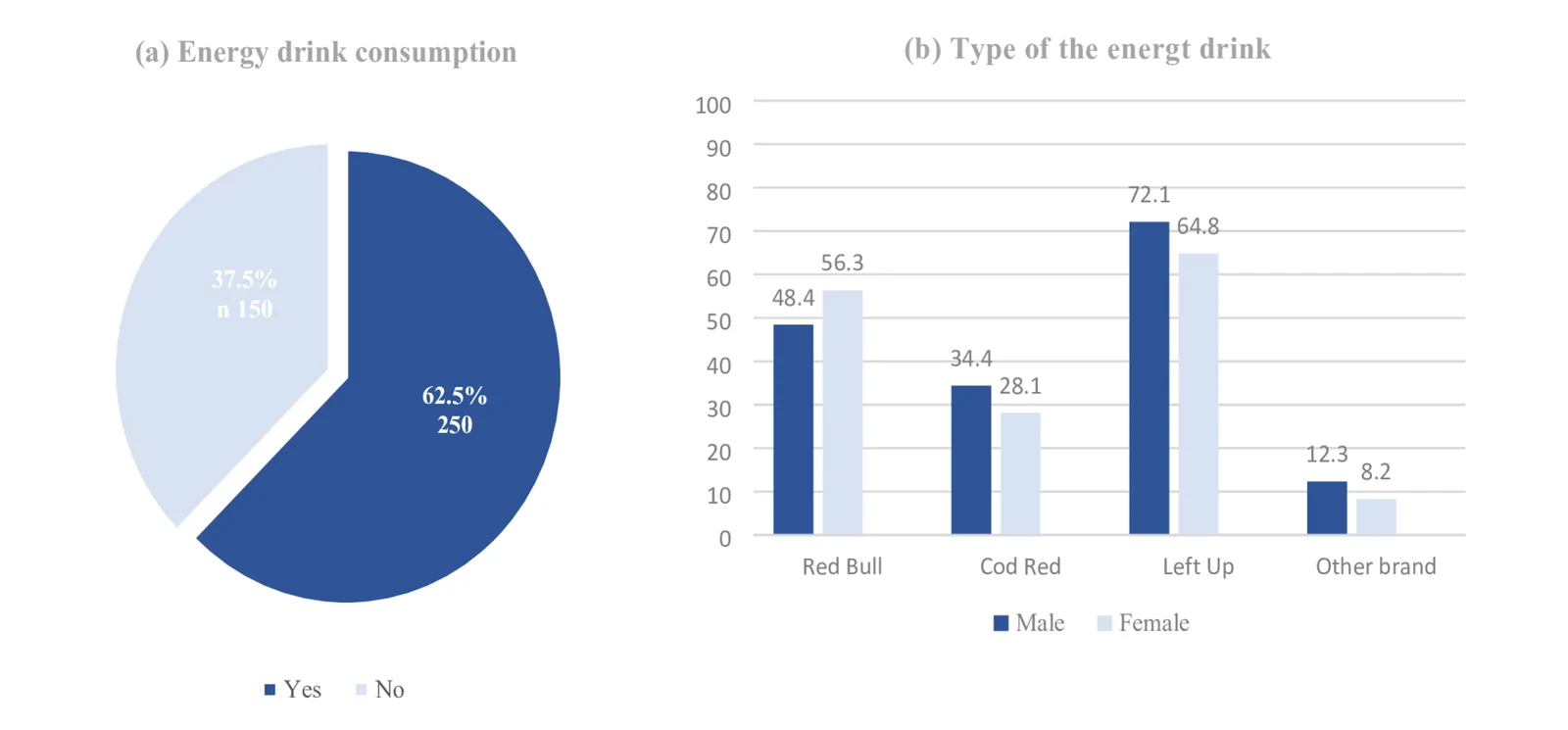
(**a**) Prevalence of current energy drink consumption among all participants (*n* = 400). **b** Distribution of commonly used energy drink types among energy drink users only (*n* = 250)

The findings indicate that the highest proportion of students were consumers of energy drinks, with 250 (62.5%) reporting current use (a). Among the types of energy drinks (b), Lift Up showed the highest preference among both males (72.1%) and females (64.8%) (Fig. 1).

The overall prevalence of energy drink consumption among participants was 62.5%. Significant associations were observed between energy drink consumption and age (*p* = 0.011), sex (*p* = 0.006), and year of study (*p* = 0.002). In contrast, no statistically significant association was found with marital status (*p* = 0.130) or field of study (*p* = 0.816). The highest consumption was observed among students aged ≤ 19 years (73.6%), males (68.5%), and second-year students (74.8%) (Table 2).

**Table 2:** Association between demographic characteristics and energy drink consumption among participants (*n* = 400)

Category	Drinking an energy drink		*P*-value
	Yes *n* (%)	No *n* (%)	
Age			
≤ 19	78 (73.6%)	28 (26.4%)	0.011
20–24	117 (60.9%)	75 (39.1%)	
≥ 25	55 (53.9%)	47 (46.1%)	
Sex			
Males	152 (68.5%)	70 (31.5%)	0.006
Female	98 (55.1%)	80 (44.9%)	
Marital Status			
Single	240 (61.9%)	148 (38.1%)	0.130
Married	10 (83.3%)	2 (16.7%)	
Field of Study			
Medical laboratories	48 (61.5%)	30 (38.5%)	0.816
Nutrition	35 (66.0%)	18 (34.0%)	
Pharmacy	37 (61.7%)	23 (38.3%)	
Dentistry	56 (61.5%)	35 (38.5%)	
Medicine	63 (65.6%)	33 (34.4%)	
Radiology	11 (50.0%)	11 (50.0%)	
level (Year)of Study			
First	38 (50.7%)	37 (49.3%)	0.002
Second	80 (74.8%)	27 (25.2%)	
Third	63 (54.8%)	52 (45.2%)	
Fourth or more	69 (67.0%)	34 (33.0%)	

Regarding consumption patterns, most participants reported frequent use of energy drinks, with 44.4% consuming them “often” and 31.2% “daily”. Evening time was the most common period of consumption (59.2%). The main reported reasons for use were reducing fatigue (70.6%) and staying awake or studying longer (70.0%). Energy drink consumption was particularly high during examination periods (76.8%). Additionally, 44.8% of participants reported concurrent use of energy drinks with other substances such as coffee, Qat, or tobacco products (Table 3).

**Table 3:** Distribution of participants’ responses to questions related to energy drink consumption patterns (*n* = 250)

Variables and Categories	Frequency (*n*)	Percentage (%)
Frequency of Consumption		
Daily	78	(31.2%)
Often	111	(44.4%)
Sometimes	61	(24.4%)
Preferred Timing of Use		
Morning	12	(4.8%)
Afternoon	90	(36.0%)
Evening	148	(59.2%)
Primary Motivations for Use*		
To stay awake / study longer	175	(70.0%)
To reduce fatigue	189	(70.6%)
To improve mood	122	(48.8%)
Habit or preference	55	(22.0%)
Social influence (friends, peer pressure, etc.)	66	(26.4%)
For the pleasant taste	130	(52.0%)
Specific High-Use Situations		
During examination periods	192	(76.8%)
While practicing sports	16	(6.4%)
Socializing with friends	42	(16.8%)

**Fig. 2:**
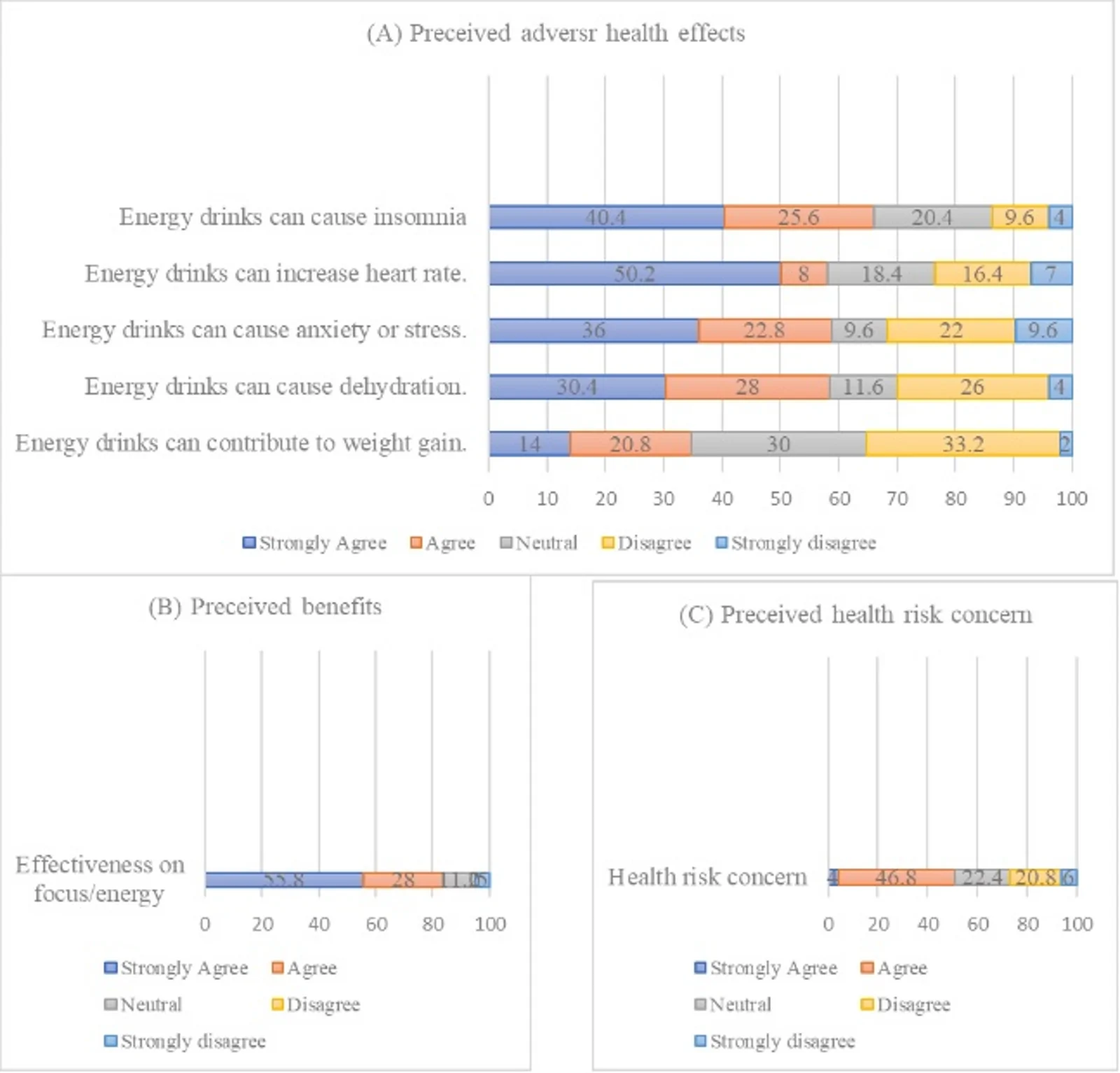
Participants’ perceptions regarding energy drink. **A** Perceived adverse health effects. **B** Perceived benefits and (**C**) Perceived health risk concerns

From the responses, participants demonstrated their highest overall level of agreement with the statement that “energy drinks are effective for improving focus and energy,” with 55.8% strongly agreeing and 28% agreeing, making a total of 83.8% overall agreement. Regarding negative health risks, participants most strongly perceived the risk of insomnia, with 66% overall agreement between those who strongly agreed and those who agreed with the statement. Anxiety/stress was next with 58.8% overall agreement, followed by increased heart rate with 58.2% overall agreement. Perceptions of dehydration risk were also high with 58.4% overall agreement, while weight gain was less strongly perceived with only 34.8% overall agreement (Fig. 2).

The students who were daily used coffee as concurrent substance with Eds was strong highly significant compared to those were often or sometimes used coffee 28 (75.7%), (*p* = 0.017). Those students who were daily chawing Qat were also statistically significant association 24 (64.9%), (*p* = 0.007) (Table 4).

**Table 4:** Co-consumption of substances according to frequency of energy drink use among co-users (*n* = 250)

Substance	Daily *n* (%)	Often *n* (%)	Sometimes *n* (%)	*P*-value
Coffee				
Yes	28 (75.7)	26 (61.9)	14 (42.4)	0.017
No	9 (24.3)	16 (38.1)	19 (57.6)	
Qat				
Yes	24 (64.9)	19 (45.2)	9 (27.3)	0.007
No	13 (35.1)	23 (54.8)	24 (72.7)	
Tobacco / Al-HOT				
Yes	12 (32.4)	11 (26.2)	6 (18.2)	0.397
No	25 (67.6)	31 (73.8)	27 (81.8)	

## Discussion

The present study was the first study conducted among students in Aden, Yemen. ED consumption was a common practice, with a consumption rate of 62.5% among the students in this study. Thiab et al. reported that the prevalence of ED consumption was 66% among university students in Jordan [31]. Unlike our findings, several studies conducted among university students reported prevalence rates of 15.2% in Italy [32], 21% in Libya [33], 24.8% in Taiwan [34], 27.2%, 29.3%, and 45.63% in Saudi Arabia [15, 35, 36], and 46.5% in Turkey [37]. Differences in cultural background, energy drink availability and marketing, academic demands, and lifestyle between regions and institutions may explain these differences. Variations in prevalence rates across studies may also be partly attributed to differences in the assessment methods and definitions used to measure energy drink consumption. In general, these studies indicate that EDs are popular among university students, but regional and institutional factors are important contributors to differences in energy drink consumption. In contrast, three studies conducted among university students in Saudi Arabia and the Caribbean revealed higher rates of ED consumption, which were 86%, 81.3%, and 74.88% [38, 39, 40]. Jacob and colleagues reported one of the highest ED consumption rates, 92%, among university students [41]. These comparable findings suggest that energy drink consumption is common among university students in several Middle Eastern countries, likely because the populations in these countries are predominantly Muslim, and the consumption of other drinks, such as alcohol, is religiously prohibited, making energy drinks the most widely accepted alternatives.

The most important finding of this study was that the rate of ED use was significantly high (73.6%, *p* = 0.011) among students in the age group ≤ 19 years. This was in line with the global trend observed in the United Arab Emirates (UAE) and Spain, where younger age groups had higher rates of ED use compared to older age groups [42, 43]. This pattern may be associated with increased study pressure and longer study hours during the early university years [43].

The current data demonstrated significantly high ED consumption among male students (68.5%, *p* = 0.006). This result was in agreement with other research from Saudi Arabia, Bangladesh, and Croatia, identifying males as strong ED consumers [44, 45, 46]. However, some studies, such as those conducted in the UAE, found no significant gender difference, possibly due to sample composition or cultural factors [42]. This may reflect differences in lifestyle patterns and exposure to stimulant beverages; however, further studies are needed to explore the underlying behavioral factors.

Regarding the study level of students in our research, the highest ED consumers were found among second-level students (74.8%), a finding supported by other studies conducted in Saudi Arabia and India [38, 47]. This suggests that the pressure to perform academically and the resulting fatigue are not confined to specific “high-intensity” majors such as medicine or engineering. Instead, the use of EDs appears to be a normalized behavioral response to the general “crunch culture” of modern higher education. Marital status was also not significantly associated with consumption (*p* = 0.130), a finding supported by other international studies [42, 43], which is likely due to the demographic homogeneity of the sample, as the vast majority of high-risk users (the ≤ 19 age group) are naturally less likely to be married.

The majority of the university students in this study, 31.2% and 44.4%, consumed energy drinks daily and frequently, respectively. This agreed with a study conducted among students at Taif University, Saudi Arabia [38]. The highest rate of ED consumption was reported in the evening (59.2%). This also corresponds to the results of international studies indicating increased consumption during periods of particularly high academic load [31, 33, 48, 49], and evening consumption is often associated with attempts to stay awake longer for studying [33, 45, 50]. Energy drinks are mainly consumed by university students to enhance academic performance and to feel alert, especially in the evening and during examination periods [9, 10, 11, 12].

The main reasons for energy drink consumption in this study were reducing fatigue and staying awake while studying. These findings suggest that academic-related demands are key drivers of consumption among students. Similar results have been reported in previous studies [45, 47, 50, 51].

For instance, the beliefs of university students regarding the health consequences of EDs, as indicated by the survey findings, showed that 58.4% of the students strongly agreed or agreed that energy drinks cause dehydration. Although dehydration is not always directly linked to ED consumption, the high caffeine and sugar content of energy drinks has the potential to cause metabolic and renal disturbances, which may contribute to dehydration [52, 53].

In terms of weight gain, only 34.8% agreed (14% strongly agreed and 20.8% agreed), but research confirms that the high sugar content of EDs contributes to obesity and type 2 diabetes [54, 55]. Anxiety or stress as a side effect of these drinks was recognized by 58.8% of respondents (36% strongly agreed and 22.8% agreed), which is consistent with research showing increased psychological distress, anxiety, and even depressive symptoms among young consumers of energy drinks [54, 55, 56]. The belief that energy drinks increase heart rate was also high (50.2% strongly agreed and 8% agreed), which is supported by research showing increased heart rate, blood pressure, and other cardiovascular symptoms such as palpitations and tachycardia [54, 57, 58].

This data recognized that 40.4% of students strongly agreed about the anosmia associated with using EDs. It was in line with the fact that sleep disturbances and poor quality of sleep are some of the most common side effects [54, 59]. Our study shows that 83.8% believe EDs were effective for focus and energy (55.8% strongly agree, 28% agree); research shows that while EDs may temporarily enhance alertness and reduce fatigue, these benefits are short-lived and often outweighed by health risks [12, 60, 61]. However, only 50.8% (4% strongly agree, 46.8% agree) express concern about health risks, despite substantial evidence of both physical and mental health risks associated with EDs use, including cardiovascular, metabolic, and behavioral problems [55, 56, 57].

The current study showed that daily coffee consumption as a concurrent substance with EDs was significantly higher among students compared with those who consumed coffee occasionally or frequently (75.7%, *p* = 0.017). This finding was consistent with previous studies conducted among university students. For example, a study by Mushkani et al. reported that higher energy drink consumption was significantly associated with daily coffee intake among university students in Kabul (*p* = 0.005) [62].

In addition, the present study found a strong significant association between daily Qat chewing and energy drink consumption (64.9%, *p* = 0.007). There is currently no previous literature involving Qat chewing among medical students consuming EDs; therefore, this finding may represent the first reported significant association among university students.

### Strengths and limitations

Our study has several strengths, including that it was the first study in Yemen to assess energy drink consumption as an important public health issue, especially among medical and health science students, thereby filling an important gap in the literature. The sample size of 400 students was adequate for a cross-sectional design and improves the reliability of the findings. The study also provides detailed data on consumption frequency, timing, reasons for use, co-ingestion with other substances such as coffee, Qat, and Al-HOT tobacco, and awareness of health risks, offering a comprehensive behavioral profile.

However, this study also has several limitations that warrant acknowledgment. First, the cross-sectional design limits the ability to establish causal relationships between energy drink consumption and associated factors, as data were collected at a single point in time. Second, the study focused on a single location and a single group, which limited the generalizability of results and insufficiently precise Eds consumption frequency. Third, the lack of multivariable logistic regression to control for confounding variables may affect interpretation of the results obtained. Fourth, the study relied on self-reported data collected through a structured questionnaire, which may be subject to recall and social desirability biases, potentially affecting the accuracy of participants’ responses regarding energy drink consumption and related behaviors. Finally, the assessment of EDs consumption in the present study was based on participants’ self-reported use through a dichotomous (yes/no) measure without specifying a time frame, which may influence prevalence estimates among students.

## Conclusions

Energy drink consumption is highly prevalent among medical students in Aden, Yemen, and is particularly common among younger and male students. The findings indicate that consumption is strongly associated with academic workload, with students primarily using energy drinks during evening study periods and examination times to manage fatigue and maintain alertness. Although many students perceive energy drinks as beneficial for improving energy and concentration, there is also a clear awareness of their potential adverse health effects, including insomnia, anxiety, dehydration, and cardiovascular symptoms.

These findings represent an important public health concern and highlight the need for targeted interventions within university settings. Universities should implement structured health education and awareness programs to inform students about the risks associated with energy drink consumption. In addition, promoting healthy study habits and stress-management strategies may help reduce reliance on such products. Policymakers and university health authorities should also consider regulating the availability and marketing of energy drinks among students to minimize potential health risks.

## Data Availability

All data generated and analyzed during this study are included in this manuscript. Additional information can be made available upon reasonable request.
